# The risk of depression and anxiety is not increased in individuals with juvenile idiopathic arthritis – results from the south-Swedish juvenile idiopathic arthritis cohort

**DOI:** 10.1186/s12969-022-00765-9

**Published:** 2022-12-09

**Authors:** Elisabet Berthold, Alma Dahlberg, Anna Jöud, Helena Tydén, Bengt Månsson, Fredrik Kahn, Robin Kahn

**Affiliations:** 1 Department of Clinical Sciences Lund, Rheumatology, Lund University, Lund, Sweden; 2 Skåne University Hospital, Lund and Malmö, Sweden; 3 Wallenberg Center for Molecular Medicine, Lund University, Lund, Sweden; 4 Department of Clinical Sciences Lund, Pediatrics, Lund University, Lund, Sweden; 5 Helsingborg Hospital, Helsingborg, Sweden; 6 Department of Laboratory Medicine, Division of Occupational and Environmental Medicine, Lund University, Lund, Sweden; 7 Department of Research and Education, Skåne University Hospital, Lund, Sweden; 8 Department of Clinical Sciences Lund, Section of Infection Medicine, Lund University, Lund, Sweden

**Keywords:** Juvenile idiopathic arthritis, JIA, Outcome, Depression, Anxiety, Mental health, Comorbidity

## Abstract

**Background:**

Children with chronic diseases are reported to have increased risk of psychiatric comorbidity. Few studies have investigated this risk in juvenile idiopathic arthritis (JIA), with conflicting results. We performed a population-based, longitudinal cohort study of the risk of depression and anxiety in south-Swedish patients with juvenile arthritis.

**Methods:**

The south-Swedish JIA cohort (*n* = 640), a population-based cohort with validated JIA diagnosis 1980 – 2010 and comparators, a reference group of 3200 individuals free from JIA, matched for sex, year of birth and residential region, was used. Data on comorbid diagnosis with depression or anxiety were obtained from the Skåne Healthcare Register, containing all healthcare contacts in the region, from 1998 to 2019. We used Cox proportional models for the calculation of hazard ratios.

**Results:**

During the study period, 1998 to 2019, 93 (14.5%) of the individuals in the JIA group were diagnosed with depression, and 111 (17.3%) with anxiety. Corresponding numbers among the references was 474 (14.8%) with depression and 557 (17.4%) with anxiety. Hazard ratio for depression was 1.1 (95% CI 0.9 – 1.5) in females and 0.8 (95% CI 0.5 – 1.4) in males, and for anxiety 1.2 (95% CI 0.9 – 1.5) in females and 0.6 (95% CI 0.4 – 1.1) in males. There were no statistically significant hazard ratios when analyzing subgroups of JIA patients with long disease duration or treatment with disease-modifying antirheumatic drugs.

**Conclusions:**

Individuals with JIA do not have any statistically increased risk of being diagnosed with depression or anxiety compared to matched references.

**Supplementary Information:**

The online version contains supplementary material available at 10.1186/s12969-022-00765-9.

## Background

Juvenile idiopathic arthritis (JIA) is a chronic inflammatory joint disease affecting children and adolescents under the age of 16, causing joint swelling with mobility limitations and pain as possible short-term and long-term consequences [1]. The mean incidence of JIA 2002 – 2010 was 12.8/100 000 children in the south-Swedish region of Skåne [2], with reported numbers internationally ranging from 3.1 – 15.0/100 000 children [3–8].

According to the definition from the International League of Associations for Rheumatology (ILAR), JIA serves as an umbrella term for seven subcategories, defined by the number of inflamed joints, additional organ involvement and presence of systemic features. Six of the subcategories have adult equivalents and only oligoarticular JIA, where the majority of patients have antinuclear antibodies (ANA) and disease onset before the age of six, is very uncommon in adults [9]. Since the subcategories of JIA includes patients with heterogenous as well as overlapping phenotypes, such as ANA-positivity, early onset and asymmetrical joint involvement, there are suggestions of a new classification system where ANA positive children with early onset of arthritis (irrespective of joint count) should be a defined subtype. This subtype is specific for arthritis in children. The suggestions also include other refinements of the subtypes to make the pediatric arthritides further resemble their adult counterparts [10, 11].

Although JIA is considered a childhood disease, 45.6% of the patients in the Nordic JIA cohort still had active disease 18 years after diagnosis [12] and 41% of the patients still had active disease or needed pharmacological treatment in a Norwegian study 30 years after diagnosis [13]. The pharmacologic treatment of JIA has evolved considerably during the last decades, especially with the introduction of biological disease-modifying antirheumatic drugs (DMARDs) in the start of the twenty-first century. Before biological DMARDs (bDMARDs) were available, other synthetic DMARDs such as gold, sulfasalazine and chloroquine phosphate were used and are today termed conventional synthetic DMARDs (csDMARDs) [1]. Methotrexate is today the most widely used csDMARD and was introduced as treatment for juvenile arthritis in Sweden in the mid 1980s.

Studies have demonstrated that children with chronic diseases are more likely to develop psychiatric comorbidities than the general population [14, 15]. In recent years more attention is given to the possible effect of inflammation for the pathophysiology of mood-disorders and anxiety. In a stressful situation, secreted stressors activate inflammatory pathways in circulating mononuclear cells, leading to increased levels of pro-inflammatory cytokines that through different mechanisms exert their effects on the central nervous system, for example by decreasing the concentration of monoamines [16, 17]. Clinical symptoms of depression have been reported in 7 – 36% of children with JIA and 7 – 64% reported significant anxiety during childhood [18].

In the study area of southern Sweden, the prevalence of depression in the general population in 2019 was 1.09%. Prevalence for anxiety was 1.04%, with female predominance (sex ratio 3:2 and 2:1 respectively) [19].

Swedish children with the chronic, autoimmune disorders childhood-onset inflammatory bowel disease (IBD), type one diabetes mellitus and celiac disease have significantly increased risk for mood-disorders and anxiety compared to the general population in long-term follow-up studies [20–22]. Since studies of psychiatric comorbidities in JIA report conflicting results, the risk of depression and anxiety for individuals with JIA is not known.

By using a population-based cohort of individuals diagnosed with JIA 1980 – 2010 with a follow-up time up to 39 years, our main aim was to investigate if individuals with JIA have increased risk of developing depression or anxiety as compared to references without JIA. We also aimed to investigate if JIA individuals are diagnosed with depression and anxiety at an earlier age as compared to sex- and age-matched references without JIA, and if the risk of depression and anxiety differed between the defined groups of JIA patients; ANA-positive disease with early onset, and DMARD treated.

## Methods

### Study design

We included 251 individuals from the previously published population-based south-Swedish JIA cohort as study material [2] and further expanded the cohort with individuals living in Skåne and diagnosed with juvenile arthritis 1980 – 2001 (*n* = 400). The additional cases were collected in accordance with the case collection process used for the published cohort [2]. In brief, registered International Classification of Diseases (ICD) codes for juvenile arthritis was made in the local clinical database at the regional centre for pediatric rheumatology, Lund University Hospital, and at the National patient register at the National Board for Health and Welfare (NBHW) using the codes: 696.00, 712, 713.10–19 and 714.93 (ICD-8); 696A, 713B and 714 (ICD-9); and M08-M09 (ICD-10). The diagnosis could be registered as primary or secondary diagnosis, both from outpatient (at Lund University Hospital) and inpatient healthcare visits anywhere in the region. The diagnosis was validated through review of medical records. Two-part consensus was used for diagnosis when a case was considered uncertain. Cases were included in the cohort if considered diagnosed before the 16^th^ birthday between 1 January 1980 and 31 December 2001 while living in Skåne. (A detailed description of the case collection process and reasons for exclusion is enclosed as Additional file 1.) Demographic data, immunological data and annually prescribed pharmacologic treatment was registered during the medical record review. Methotrexate, chloroquine phosphate, sulfasalazine, azathioprine, gold, mycophenolate, cyclosporine, penicillamine, and chlorambucil was registered as csDMARDs, and tumor necrosis factor (TNF)-inhibitors, anakinra, tocilizumab, and ustekinumab, as bDMARDs.

Together these individuals now constitute the south-Swedish JIA cohort and includes 651 individuals diagnosed with juvenile arthritis 1980 – 2010 while living in Skåne, the southernmost region of Sweden. For every individual, five individuals were collected as reference population from the Skåne Healthcare Register (SHR) (described in the section below) and were matched for year of birth, sex, and residential region. The study period was 1998 – 2019 and cases and references were followed until migration, death, diagnosis with depression or anxiety, or end of study period, whichever occurred first. Eleven of the individuals in the JIA cohort did not have a registered healthcare visit in Skåne during the study period and were excluded from the cohort for this study. The study population therefore consists of 640 individuals with JIA and 3200 references without JIA.

The study was conducted according to the declaration of Helsinki and was approved by the Regional Ethical Review Board for southern Sweden (2011/379, 2013/192 and 2015/62) and the National Ethical Review Agency (2020–02935).

### Study area

Study area was Skåne, the southernmost region in Sweden consisting of 33 municipalities with an area of 11 027 km^2^ [23]. The population was 1 377 827 (13.3% of the Swedish population) by December 2019, with children 0 – 15 years constituting 19.4% of the residents [24]. In the study area, there are four hospitals providing inpatient psychiatric care. Psychiatric outpatient clinics are situated at an additional eight locations. Pediatric psychiatric outpatient care is available in eleven cities as well as in 177 primary care centers, where milder psychiatric conditions are treated.

### Data source

Sweden has a publicly financed healthcare system, and care is subsidized for children. Swedish citizens are given a unique twelve-digit identity number which is used as identification and allows linkages between healthcare and administrative registers.

Skåne Healthcare Register (SHR) is a regional administrative healthcare register that includes registered ICD codes for healthcare visits in Skåne since 1998. At start predominately only inpatient care and outpatient visits were registered, but since 2004 all care, including primary care, is included. Appointments with caregivers other than physicians are available since 2004. SHR covers all levels of care, with data showing that 97% of the general population at some point during 2012–2016 had at least one health-care contact [25].

We acquired ICD codes F32 (depressive disorder), F33 (recurring depression), F34.1 (dysthymia) and F41 (anxiety) according to ICD-10 1998 – 2019. To decrease the risk of including improperly registered diagnosis codes, one registered code at an inpatient visit, or two codes registered at separate outpatient visits was considered as a verified depression or anxiety diagnosis.

### Statistical analysis

Demographics was described with descriptive statistics. Potential differences in age at depression/anxiety diagnosis between the cohort and references were investigated using Mann–Whitney U-test, where a p-value of < 0.05 was considered significant. The frequency of diagnosis is presented as cumulative incidence, where the numbers include all registered diagnosis regardless of where the patient was treated i.e., primary-, psychiatric-, or somatic care.

Conditional Cox proportional hazard regression models were used for calculation of hazard ratios (HR) with 95% confidence interval (CI) for depression and anxiety respectively, stratified on matched sets for sex and year of birth. Days from JIA diagnosis/cohort inclusion was used as time variable. HR was calculated for females and males separately. Individuals diagnosed with depression (*n* = 1 for JIA and *n* = 5 for references) or anxiety (*n* = 2 for JIA and *n* = 8 for references) before onset of JIA were excluded from the analysis. Information on date of JIA diagnosis was missing in five JIA cases and therefore these cases, and their corresponding 25 references, were not included in the analysis.

Statistical analyses were performed using readxl, dplyr, lubridate, survival, survminer in R 3.6.2 software (R Foundation for Statistical Computing; https://www.r-project.org/), openepi.com [26], and SPSS® 27.0 for Macintosh.

## Results

### Demographics

Two thirds of the individuals were female. The median age at JIA diagnosis was 8.7 years (interquartile range (IQR) 3.7 – 12.7 years) independent of decade for JIA diagnosis. Approximately 50% were ANA-positive and 30% of the JIA patients were diagnosed with ANA-positive disease with onset before the age of six. Subtype distribution according to ILAR definition is enclosed as Additional file 2. Median follow-up time to depression was 13.3 years for females and 15.1 years for males, and 13.4 vs 15.0 years for anxiety.

In the total cohort, 53.8% were at some point during their disease course treated with csDMARDs (methotrexate 42.4%) and 54.4% with any DMARD (Table 1).

**Table 1:** Demographics and pharmacologic treatment in the JIA-cohort and references

Characteristics	JIA (*n* = 640)	References (*n *= 3200)
Female (n (%))	433 (67.7)	2165 (67.7)
Age at JIA-diagnosis and/or cohort entry, yrs (median (IQR))	8.7 (3.7—12.7) n 633	8.7 (3.7—12.7)
ANA positive, early onset (n (%))	192 (30.0)	NA
< 6 years at diagnosis (n (%))	255 (39.8)	1275 (39.8)
ANA positive (n (%))	326 (50.9)	NA
Methotrexate (n (%))	270 (42.2)	NA
csDMARDs (n (%))	344 (53.8)	NA
bDMARDs (n (%))	119 (18.6)	NA
Any DMARD (n (%))	348 (54.4)	NA
Depression (n (%))	93 (14.5)	474 (14.8)
Anxiety (n (%))	111 (17.3)	557 (17.4)

*Abbreviations: IQR* interquartile range, *ANA* antinuclear antibody, *DMARD* disease-modifying antirheumatic drug, *csDMARD* conventional synthetic DMARD, *bDMARD* biological DMARD, *NA* not applicable

### Frequency of diagnosis

During the study period, 1998 through 2019, 93 (14.5%) individuals with JIA were diagnosed with a depressive disorder and 111 (17.3%) with an anxiety disorder. Corresponding numbers in the reference group was 474 (14.8%) for depression and 557 (17.4%) for anxiety. Sex ratio female to male was 4:1 for depression and 5:1 for anxiety in the JIA cohort, in comparison to 3.6:1 for depression and 3.3:1 for anxiety in the reference group. The median age for depression in the JIA cohort was 23.5 years and 23.6 years for anxiety. This was not different compared to references, 24.1 years and 23.7 years for depression and anxiety, respectively (*p* = 0.4586 and *p* = 0.5413, Mann–Whitney U-test). During 1998 – 2010, 11.8% in the JIA group received a diagnosis of depression and 16.2% a diagnosis of anxiety ≤ 18 years of age. A table presenting demographics and pharmacologic treatment in the JIA group divided by presence of depression and anxiety is enclosed as Additional file 3.

Both in the depression and anxiety analyses 68 individuals were lost to follow-up (18 JIA, 50 references) and two references died during the study period.

### Cox proportional hazards

Hazard ratio for depression was 1.1 (95% CI 0.9 – 1.5) for females and 0.8 (95% CI 0.5 – 1.4) for males. HR for anxiety was 1.2 (95% CI 0.9 – 1.5) for females and 0.6 (95% CI 0.4 – 1.1) for males.

**Fig. 1:**
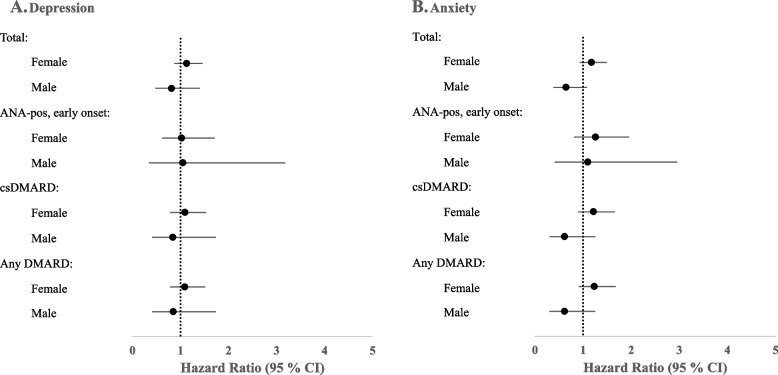
Hazard ratios for depression and anxiety in patients with JIA compared with references without JIA. Conditional Cox proportional hazard regression models were used for the calculation of hazard ratios (HR) with 95% confidence interval (CI) for **A** Depression (ICD codes F32, F33 and F34.1) and **B** Anxiety (ICD code F41), stratified on matched sets for sex and year of birth. Patients in the JIA cohort were compared to the reference group in all analyses and the JIA cohort was further divided into subgroups of ANA-positive disease with onset before the age of six, and treatment with conventional synthetic DMARDs (csDMARDs) or any DMARD. The bars indicate 95% CIs with markers for HR. No significant difference was found in the analysis

Neither analysis of JIA subgroups with ANA-positive disease with onset before the age of six, treatment with csDMARDs, nor treatment with any DMARD compared to matched references, showed any statistically significant HR (Fig. 1). To further validate that lack of comorbid ICD codes from 1980 to 1998 did not influence the result, additional analyses were repeated among individuals diagnosed with JIA between 1998 and 2010. Here, males with JIA had a significant decreased HR 0.4 (95% CI 0.1 – 0.9) for anxiety compared to references when looking at the total group (*p* = 0.04), but no difference was found in the other analyses (figure showing the results from these analyses is enclosed as Additional file 4).

**Fig. 2:**
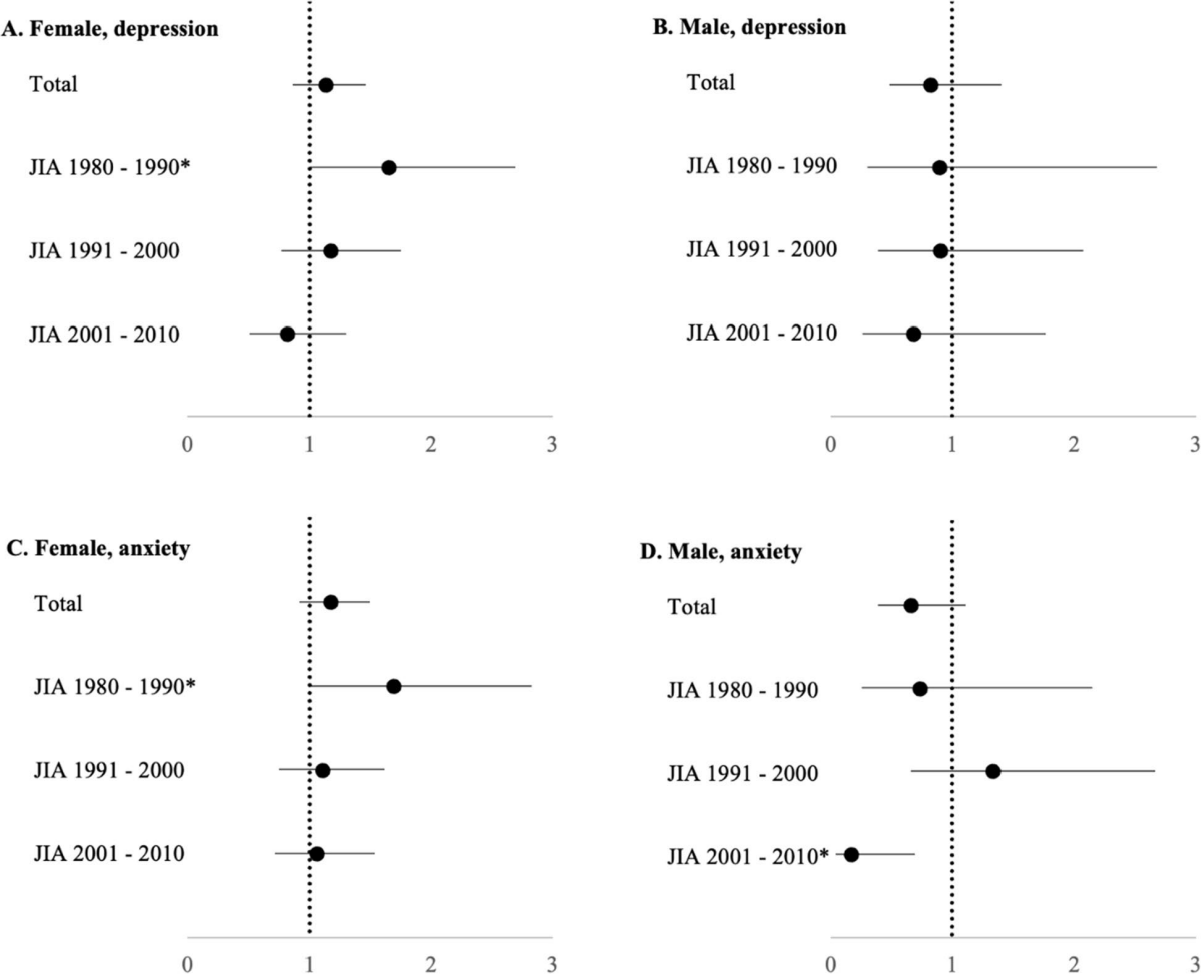
Hazard ratios for depression and anxiety in patients with JIA compared with references without JIA, demonstrated in periods of JIA diagnosis. Conditional Cox proportional hazard regression models were used for the calculation of hazard ratios (HR) with 95% confidence interval (CI) for depression (ICD codes F32, F33 and F34.1) and anxiety (ICD code F41), stratified on matched sets for sex and year of birth. Patients in the JIA cohort were compared to the reference group and the individuals were further divided based on year of JIA diagnosis/inclusion. The bars indicate 95% CIs with markers for HR. Females with JIA diagnosed 1980 – 1990 had significantly increased HR for depression HR 1.7 (95% CI 1.02 – 2.83) as well as anxiety HR 1.7 (95% CI 1.01 – 2.83) (* = *p* < 0.05). Males with JIA diagnosed 2001 – 2010 had significantly decreased HR for anxiety HR 0.2 (95% CI 0.04 – 0.67) (* = *p* < 0.05)

We chose to further analyze possible differences in HR by dividing the individuals by year of JIA diagnosis/study inclusion, with the subgroups roughly representing different treatment eras (1980 – 1990 “pre-DMARDs”, 1991 – 2000 “methotrexate”, 2001 – 2010 “bDMARDs”). Females with JIA diagnosed 1980 – 1990 had significant increased HR 1.7 (95% CI 1.02 – 2.83) for depression and HR 1.7 (95% CI 1.01 – 2.83) for anxiety compared to references. Males with JIA diagnosed 2001 – 2010 had significant decreased HR 0.2 (95% CI 0.04 – 0.67) for anxiety compared to references (Fig. 2).

## Discussion

In this study we showed that individuals with JIA in our population based south-Swedish cohort did not have an increased risk of being diagnosed with depression or anxiety compared to age- and sex matched references. Neither did subgroup-analyses, based on disease onset or DMARD treatment, show any increased risk.

Other studies have shown that children with chronic diseases, inflammatory as well as non-inflammatory, are more likely to develop psychiatric comorbidities than the general population [14, 15, 20–22]. There was therefore reason to believe that this also applied to JIA. Indeed, a chronic pediatric disease may serve as a stressor as the child or adolescent must cope with managing the disease alongside with normal bodily and behavioural changes growing up and since JIA often has a remitting course, also in periods of disease remission patients are psychologically under the threat of a flare. In addition, the cardinal symptom of JIA is arthritis, a condition causing pain and limited motion. For some patients, systemic symptoms of inflammation such as fatigue and malaise are also present. Pain and fatigue are factors known to negatively affect quality of life [18, 27], possibly in part due to reduced physical activity and social exclusion. Presence of depressive symptoms can also further aggravate pain and disability in JIA [28]. However, possible protective factors may be present in our study-cohort compared to in individuals without JIA, such as team-centred care for patients with rheumatic diseases comprising preventive psychologic counselling to develop coping strategies for a life with chronic disease and easy access to psychological support in times of despair. A rheumatologist might also not register a diagnosis of depression or anxiety in a patient expressing symptoms of malaise or unease, rather interpreting this as crisis reaction after diagnosis or an effect of chronic pain, which differs in individuals without a chronic diagnosis. Our results do not exclude the possibility of individuals with JIA having more distress symptoms or that the potential impact of these symptoms have greater impact on their quality of life than in the references population, since our study does not include patient reported data and symptoms. Follow-up of the psychological well-being should still be carried out in the JIA patient group.

Pediatric rheumatologists today work with “treat-to-target” strategies with frequent visits and treatment adjustments to achieve remission as fast as possible. The goal is to keep periods with inflammatory activity short to prevent adverse somatic and psychiatric effects. Prior to the introduction of methotrexate and bDMARDs, the inflammatory activity in JIA was often difficult to effectively reduce. One could speculate that modern and more “aggressive” treatment strategy influences the risk of psychiatric co-morbidities, as it is suggested that chronic inflammation may serve as a stressor for psychiatric disease [16, 17]. It would have been interesting to compare measures of disease activity to the presence of depression or anxiety in our study, but unfortunately, we did not have active joint count and laboratory markers of inflammation in all cases. We did however choose to study the risk of depression and anxiety in subgroups of individuals in need of DMARD treatment, since this may serve as surrogate marker for more severe disease and high inflammatory activity, but in neither of these subpopulations the risk was different compared to matched references. However, there are other reasons than high inflammatory activity to start DMARD treatment, and the lack of a good marker for disease activity is a limitation to our study.

The subgroup of ANA-positive disease with early onset is especially interesting as this diagnostic subgroup is believed to have unique pathophysiological mechanisms and is typical for pediatric onset arthritis. Many of the patients with this subtype have oligoarticular disease, which is associated with good prognosis of remission and treatment response [1]. Nevertheless, with symptom debut before the age of six, these patients have the longest disease duration, which was associated with the development of psychiatric illness in the study by Kyllönen et al. [29]. However, we did not find such an association in our study and there was no difference in median age at diagnosis with depression or anxiety between individuals with JIA and references, which further strengthens that disease duration have little impact to the risk for depression or anxiety in JIA.

The finding of increased HR for depression and anxiety in the group of individuals with diagnosis/study inclusion 1980 – 1990 should be interpreted with caution since it might have other explanations than change of treatment regime. We have studied the cumulative incidence and since the risk of being diagnosed with depression or anxiety increases with age and there is greater awareness of depression and anxiety in the general population as well as at centres for primary care 2019 compared to 1998, the oldest individuals in this study are the ones with the highest risk of comorbid diagnosis. Another possible explanation to why the HR decreases over time in, at least in the female subgroups, is that symptoms of depression and anxiety might be interpreted as a reaction to the JIA diagnosis during the first years of disease, while they are considered and treated as a true diagnosis later in the disease course.

Our study has some limitations. In the creation of the cohort, we only had full regional coverage of inpatient visits because registration of ICD codes for outpatient visits were not mandatory in Sweden until 2002. We had access to outpatient visits from Lund University Hospital (2010 renamed as Skåne University Hospital) for the total study period. Thus, there is the potential risk of us having missed to include JIA cases with mild disease diagnosed prior to 2002 with only outpatient visits at healthcare facilities other than the Lund University Hospital. However, we believe that number to be limited since most children needed inpatient care for rehabilitation and treatment in the 1980’s, and intra-articular corticosteroid injections in younger children were administered during inpatient care in the 1980’s and 90’s.

We only had access to registered ICD codes for depression and anxiety since 1998. This may result in a possible overestimation of incident cases since we did not exclude individuals that might have had such a diagnosis before the study period and hence have an increased risk of a repeated diagnosis. The references are selected on having at least one healthcare consultation during follow-up. Thus, we might also overestimate the existence of depression and anxiety in the control group. The lack of data may on the other hand underestimate the frequency of psychiatric comorbidities since we lack 18 years of data. The subgroup analysis among the individuals diagnosed with JIA between 1998 – 2010 did in fact find a decreased HR for anxiety in JIA males, as in the subgroup analysis based on year of JIA diagnosis 2001—2010. However, the cases are few, CI is wide and just below the significance level, and the clinical relevance of this finding is thus questionable.

The SHR did not have full coverage of diagnosis codes registered in outpatient care, mainly from primary care, until 2004 which also contributes to the risk of underestimation. However, the presence of depression and anxiety as well as the cumulative incidence of these conditions in our study is higher, both in the JIA cohort and references, than reported numbers in other Swedish studies on comorbid psychiatric disorders in childhood-onset IBD (6.6% mood disorders and 10.4% anxiety) and celiac disease (6.2% mood disorders and 8.2% anxiety) [20, 21]. In these studies, they considered one registered ICD code as a true comorbid event and did also not validate the diagnosis through medical record review or personal interview. Therefore, the requirement in our study for two codes if registered only in outpatient care would rather have reduced the number of cases. However, the above-mentioned studies had shorter median follow-up time, 9 and 12.2 years respectively, and did not have access to ICD codes registered from Swedish primary care facilities, where mild and moderate variants of depression and anxiety often are treated. Our increased presence and cumulative incidence can partly be explained by the longer follow-up time (13.3 years for females and 15.1 years for males) and inclusion of primary care diagnoses, at least since 2004.

There are also strengths to our study. We have a population-based cohort with validated diagnoses of JIA in a well-defined region of Sweden, thus including all severities of the disease from mild and self-limiting to remitting and functional disabling disease course compared to the Finnish study by Kyllönen et al. [29]. Most other published studies on mental health in JIA patients have cross-sectional study design and patient reported outcome [18, 28, 30]. Our study is longitudinal with follow-up up to 39 years after JIA onset and based on diagnosis assessed by medical care givers.

## Conclusions

In conclusion, we have studied the long-term risk of depression and anxiety in a south-Swedish population-based cohort of individuals with JIA diagnosed 1980 – 2010. This is, to our knowledge, the largest study with longitudinal follow-up of psychiatric comorbidities in JIA. Individuals with juvenile arthritis do not appear to be diagnosed with depression or anxiety at an earlier age or more often than sex- and age-matched references. We neither found any increased risk in subgroups with longer disease duration or more severe disease. Thus, we conclude that growing up with inflammatory arthritis do not appear to increase the risk of being diagnosed with depression or anxiety disorders later in life.

### Supplementary Information

**Additional file 1.** “Case collection process”. A flowchart of included cases and reasons for exclusion.

**Additional file 2.** “Subtype distribution according to ILAR definition”. A table of the subtype distribution according to the revised ILAR classification criteria from 2001.

**Additional file 3.** “Demographic information and pharmacologic treatment presented in groups based on presence of depression or anxiety”. A table including the same demographic information as in table 1, divided by the presence of depression and anxiety respectively.

**Additional file 4.** “Hazard ratios of JIA patients and references included 1998 – 2010”. A figure demonstrating hazard ratios with 95% confidence interval for depression and anxiety as in figure 1, but only including individuals diagnosed with JIA/included as reference between 1 January 1998 to 31 December 2010.

## Data Availability

The datasets generated and analyzed during this current study are not publicly available as they contain information that could compromise research participant privacy. The data are available and anonymized from the corresponding author (EB) on reasonable request and appropriate permission from regulatory authorities.

## Abbreviations

JIA: Juvenile idiopathic arthritis
ILAR: International league of associations for rheumatology
ANA: Antinuclear antibodies
DMARD: Disease-modifying antirheumatic drug
bDMARD: Biological disease-modifying antirheumatic drug
csDMARD: Conventional synthetic disease-modifying antirheumatic drug
IBD: Inflammatory bowel disease
NBHW: National board for health and welfare
ICD: International classification of diagnosis
EULAR: European league against rheumatism
SHR: Skåne healthcare register
HR: Hazard ratio
CI: Confidence interval
IQR: Interquartile range
