# Correction: The risk of depression and anxiety is not increased in individuals with juvenile idiopathic arthritis – results from the south-Swedish juvenile idiopathic arthritis cohort

**DOI:** 10.1186/s12969-024-00995-z

**Published:** 2024-06-17

**Authors:** Elisabet Berthold, Alma Dahlberg, Anna Jöud, Helena Tydén, Bengt Månsson, Fredrik Kahn, Robin Kahn

**Affiliations:** 1 Department of Clinical Sciences Lund, Rheumatology, Lund University, Lund, Sweden; 2 Skåne University Hospital, Lund and Malmö, Sweden; 3 Wallenberg Center for Molecular Medicine, Lund University, Lund, Sweden; 4 Department of Clinical Sciences Lund, Pediatrics, Lund University, Lund, Sweden; 5 Helsingborg Hospital, Helsingborg, Sweden; 6 Department of Laboratory Medicine, Division of Occupational and Environmental Medicine, Lund University, Lund, Sweden; 7 Department of Research and Education, Skåne University Hospital, Lund, Sweden; 8 Department of Clinical Sciences Lund, Section of Infection Medicine, Lund University, Lund, Sweden

**Correction** **:** **Pediatr Rheumatol 20, 114 (2022)**

**https://doi.org/10.1186/s12969-022-00765-9**

Following publication of the original article [1], we have been notified of minor deviations in the Cox analyses in Figure 2, due to seven controls that lacked date for loss to follow-up. None of the conclusions of the article is altered by this error and all significances remain the same.

Originally published Figure 2


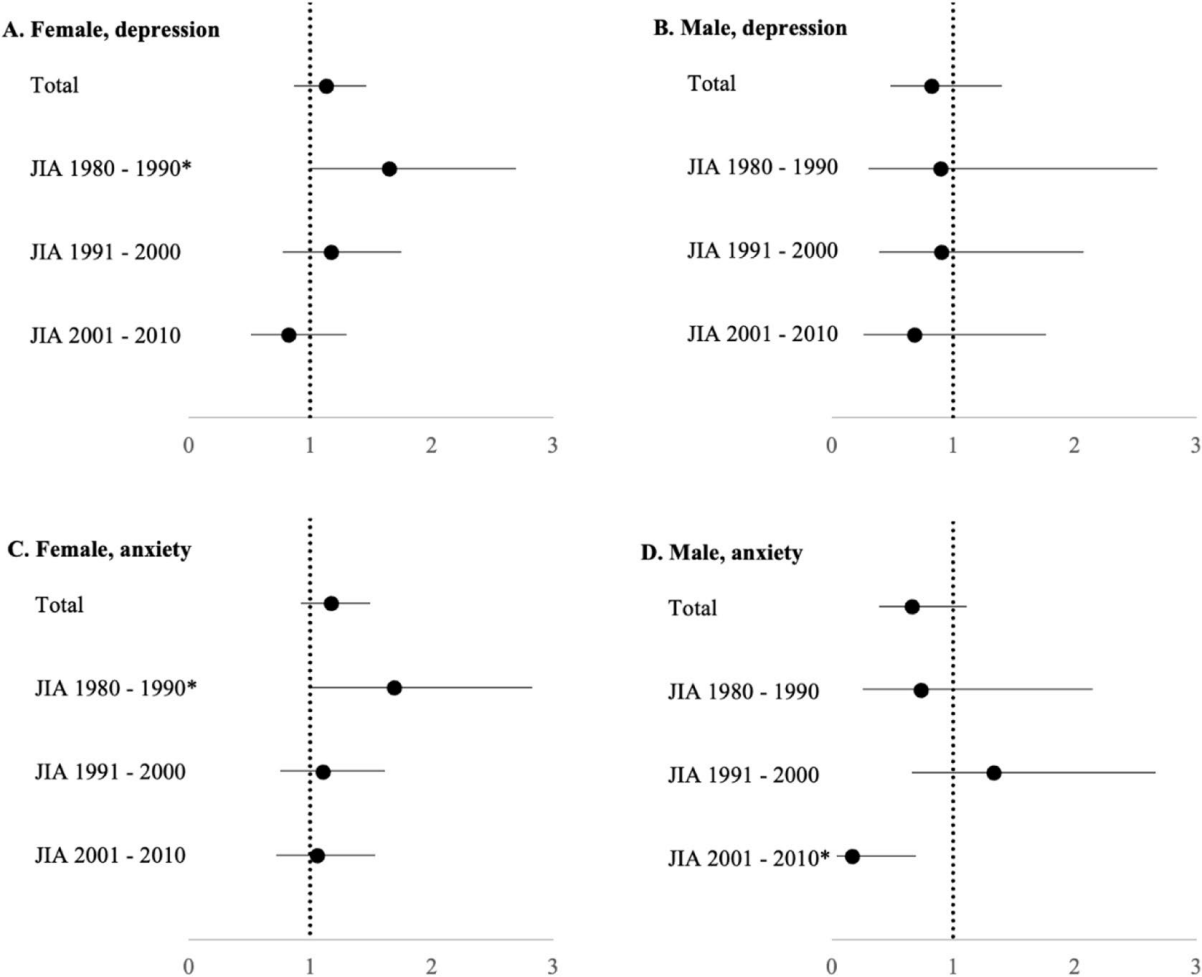


Corrected Figure 2:


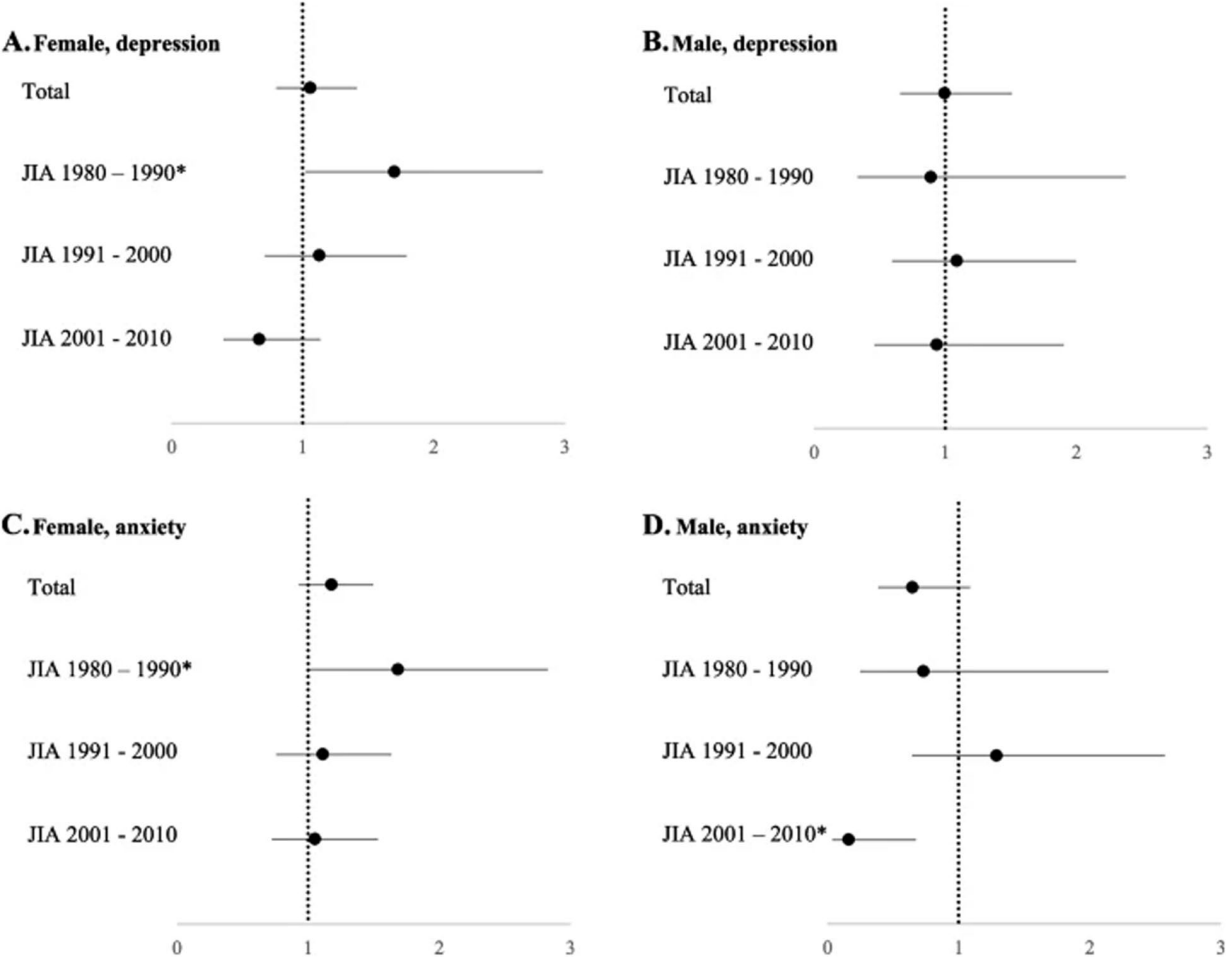


The original article has been corrected.
