# Supplementary material for: The risk of depression and anxiety is not increased in individuals with juvenile idiopathic arthritis – results from the south-Swedish juvenile idiopathic arthritis cohort
Source: Pediatr Rheumatol Online J. 2022 Dec 9;20:114. doi: 10.1186/s12969-022-00765-9 (PMC9733298; doi:10.1186/s12969-022-00765-9)
Supplement: Supplementary file 1 — Additional file 1. “Case collection process”. A flowchart of included cases and reasons for exclusion. [file 12969_2022_765_MOESM1_ESM.docx]

**Additional file 1: Case collection process**

Patients included in the cohort were to be diagnosed with juvenile arthritis 1980 – 2010 in Skåne before the age of 16. International classification of diseases (ICD) codes for juvenile arthritis were collected from the local hospital register and the National Board for Health and Welfare (NBHW) (696.00, 712, 713.10-19 and 714.93 (ICD-8); 696A, 713B and 714 (ICD-9); and M08-M09 (ICD-10)). The cases were excluded due to the reasons stated above. Eleven additional individuals were excluded from the analysis due to not having had healthcare visits registered in Skåne Healthcare Register during the study period 1998 – 2019.
