# Supplementary material for: The risk of depression and anxiety is not increased in individuals with juvenile idiopathic arthritis – results from the south-Swedish juvenile idiopathic arthritis cohort
Source: Pediatr Rheumatol Online J. 2022 Dec 9;20:114. doi: 10.1186/s12969-022-00765-9 (PMC9733298; doi:10.1186/s12969-022-00765-9)
Supplement: Supplementary file 2 — Additional file 2. “Subtype distribution according to ILAR definition”. A table of the subtype distribution according to the revised ILAR classification criteria from 2001. [file 12969_2022_765_MOESM2_ESM.docx]

**Additional file 2: Subtype distribution according to International League of Associations for Rheumatology (ILAR) criteria**

| **ILAR subtype** | n = 640 (%) |
| --- | --- |
| sJIA | 27 (4.2) |
| Oligo | 326 (50.9) |
| RF- poly | 105 (16.4) |
| RF+ poly | 36 (5.6) |
| ERA | 47 (7.3) |
| JPsA | 46 (7.2) |
| uJIA | 53 (8.3) |

Abbreviations: sJIA – systemic JIA, Oligo – oligoarthritis, RF- poly – rheumatoid factor negative polyarthritis, RF+ poly – rheumatoid factor positive polyarthritis, ERA – enthesitis related arthritis, JPsA – juvenile psoriatic arthritis, uJIA – undifferentiated arthritis

A table of the subtype distribution according to the revised ILAR classification criteria from 2001. Since the cohort also includes individuals diagnosed according to EULAR definition for juvenile chronic arthritis, the subtype for these patients was adapted to the ILAR definition at study inclusion according to the information stated in the medical review.
