# Supplementary material for: The risk of depression and anxiety is not increased in individuals with juvenile idiopathic arthritis – results from the south-Swedish juvenile idiopathic arthritis cohort
Source: Pediatr Rheumatol Online J. 2022 Dec 9;20:114. doi: 10.1186/s12969-022-00765-9 (PMC9733298; doi:10.1186/s12969-022-00765-9)
Supplement: Supplementary file 3 — Additional file 3. “Demographic information and pharmacologic treatment presented in groups based on presence of depression or anxiety”. A table including the same demographic information as in table 1, divided by the presence of depression and anxiety respectively. [file 12969_2022_765_MOESM3_ESM.docx]

**Additional file 3: Demographic information and pharmacologic treatment presented in groups based on presence of depression or anxiety**

| Characteristics | JIA (n=640) | JIA with depression (n=93) | JIA with anxiety (n=111) | JIA without depression/anxiety (n=445) | References (n=3200) |
| --- | --- | --- | --- | --- | --- |
| Female (n (%)) | 433 (67.7) | 76 (81.7) | 94 (84.7) | 287 (64.5) | 2165 (67.7) |
| Age at JIA-diagnosis and/or cohort entry,  y (median (IQR)) | 8.7 (3.7 - 12.7) n 633 | 10.4 (4.3 – 13.2) | 9.3 (3.8 – 12.9) | 7.7 (3.2 – 12.5) | 8.7 (3.7 - 12.7) |
| ANA positive, early onset (n (%)) | 192 (30.0) | 23 (24.7) | 32 (28.8) | 148 (33.3) | NA |
| < 6 years at diagnosis (n (%)) | 255 (39.8) n 633 | 31 (33.3) | 43 (38.7) | 194 (43.6) n 444 | 1275 (39.8) |
| ANA positive (n (%)) | 326 (50.9) | 46 (49.5) | 59 (53.2) | 231 (51.9) | NA |
| Methotrexate (n (%)) | 270 (42.2) | 36 (38.7) | 48 (43.2) | 186 (41.8) | NA |
| csDMARDs (n (%)) | 344 (53.8) | 55 (59.1) | 65 (58.6) | 233 (52.4) | NA |
| bDMARDs (n (%)) | 119 (18.6) | 21 (22.6) | 29 (26.1) | 80 (18.0) | NA |
| Any DMARD (n (%)) | 348 (54.4) | 56 (60.2) | 67 (60.4) | 235 (52.8) | NA |

Abbreviations: IQR – interquartile range; ANA – antinuclear antibody; DMARD - disease-modifying antirheumatic drug; csDMARD – conventional synthetic DMARD; bDMARD – biological DMARD; NA – not applicable

A table including the same demographic information and pharmacologic treatment options as in table 1, divided in groups based on the presence of depression and anxiety respectively.
