# Supplementary material for: The risk of depression and anxiety is not increased in individuals with juvenile idiopathic arthritis – results from the south-Swedish juvenile idiopathic arthritis cohort
Source: Pediatr Rheumatol Online J. 2022 Dec 9;20:114. doi: 10.1186/s12969-022-00765-9 (PMC9733298; doi:10.1186/s12969-022-00765-9)
Supplement: Supplementary file 4 — Additional file 4. “Hazard ratios of JIA patients and references included 1998 – 2010”. A figure demonstrating hazard ratios with 95% confidence interval for depression and anxiety as in figure 1, but only including individuals diagnosed with JIA/included as reference between 1 January 1998 to 31 December 2010. [file 12969_2022_765_MOESM4_ESM.docx]

**Additional file 4: Hazard ratios of JIA patients and references included 1998 - 2010**

**Hazard ratios for depression and anxiety in patients with JIA compared to sex- and age-matched references, diagnosed with JIA/included 1 January 1998 to 31 December 2010**

Conditional Cox proportional hazard regression models were used for the calculation of hazard ratios (HR) with 95% confidence interval (CI) for A) Depression (ICD codes F32, F33 and F34.1) and B) Anxiety (ICD code F41), stratified on matched sets for sex and year of birth. Patients in the JIA cohort were compared to the reference group in all analyses and the JIA cohort was further divided into subgroups of ANA-positive disease with onset before the age of six and treatment with conventional synthetic DMARDs (csDMARDs) or any DMARD. The bars indicate 95% CIs with markers for HR. The asterisk indicates that HR for anxiety in males in the total group was significant, p=0.04.
